# The measurement of media literacy in eating disorder risk factor research: psychometric properties of six measures

**DOI:** 10.1186/s40337-016-0116-0

**Published:** 2016-11-14

**Authors:** Siân A. McLean, Susan J. Paxton, Eleanor H. Wertheim

**Affiliations:** School of Psychology and Public Health, La Trobe University, Melbourne, 3086 Australia

**Keywords:** Body dissatisfaction, Disordered eating, Media literacy, Realism scepticism, Critical thinking, Reliability, Validity, Factor structure

## Abstract

**Background:**

Evaluation of media literacy-based interventions for the prevention of eating disorder risk is limited by the lack of appropriate measures with established psychometric properties with which to assess change in media literacy. This study aims to fill this gap by examining the psychometric properties and use in eating disorders risk factor research of six measures of media literacy that assess media processing and critical thinking about general media and critical thinking about appearance-focused media.

**Methods:**

The factor structure, internal consistency, test-retest reliability, and construct validity, including convergent and discriminant validity, were examined in six measures in two samples of early adolescent females. The measures were the Realism Scepticism, Similarity Scepticism, and Desirability Scepticism subscales of the Media Attitudes Questionnaire, the Fake subscale of the Critical Processing of Beauty Images Scale, the Critical Thinking about Media Messages scale, and Critical Thinking about Media Messages - Appearance Focus scale.

**Results:**

The factor structure of the measures was supported with factor analysis. Items from the Media Attitudes Questionnaire loaded on the three subscales Realism Scepticism, Similarity Scepticism, and Desirability Scepticism and items from each of the Fake subscale, Critical Thinking about Media Messages scale and Critical Thinking about Media Messages - Appearance Focus scale loaded on one scale. In addition, scores on the measures were reliable (adequate internal consistency and test-retest reliability) and valid (adequate construct, convergent, and discriminant validity) in early adolescent females. Two exceptions were Realism Scepticism, which had slightly low test-retest reliability, and the Fake subscale, for which support for construct validity was lacking.

**Conclusions:**

This study provides evidence to support the use of select media literacy measures, particularly the Realism Scepticism subscale and the Critical Thinking about Media Messages scale, in eating disorder risk factor research and the findings will contribute to enhanced evaluation of media literacy-based prevention interventions.

## Plain English Summary

Measuring outcomes of media literacy programs for eating disorders prevention is limited because the value of questionnaires to assess levels of media literacy has not been verified. This study seeks to measure adolescent girls’ levels of media literacy with six different self-report questionnaires. This will determine which questionnaires are appropriate to use in eating disorders research. The results of the study found that the questionnaires were reliable and valid and measured different aspects of media literacy. These aspects were critical thinking about media and scepticism towards media portrayals. The study findings support the use of the Realism Scepticism subscale and the Critical Thinking about Media Messages scale in future eating disorder research.

## Background

Media literacy education has been promoted as a promising approach for the prevention of eating disorders and associated risk factors, body dissatisfaction and disordered eating [1]. This approach, which teaches critical thinking about messages portrayed in media, particularly focusing on the unrealistic nature of media images, has the potential to reduce the credibility and persuasive influence of media messages that promote the thin-ideal for appearance. In line with sociocultural theories of the development of body dissatisfaction and disordered eating [2], exposure to media and associated perceived pressures to attain the thin-ideal appearance lead to body dissatisfaction and disordered eating behaviour via the mediating processes of internalising the thin media ideal (accepting and endorsing the sociocultural thin-ideal appearance as one’s personal standard) and appearance comparison (frequently comparing one’s appearance to others). Theoretically, it has been proposed that teaching skills to resist persuasive media influence and pressure prevents internalisation of the thin-ideal and appearance comparison, in turn preventing body dissatisfaction and disordered eating behaviours [3].

Tests of the role of media literacy in this field are limited by a lack of measures with established psychometric properties to assess media literacy. Although measures of media literacy have been developed in relation to health risk behaviours such as smoking [4] and tobacco and alcohol consumption [5], the psychometric properties of media literacy measures used in eating disorder risk factor research, in the main, have not been examined (e.g., [6, 7]). To advance media literacy-based eating disorder prevention, measures of media literacy with reliable and valid scores need to be identified, available, and simple to implement.

Two constructs, media processing and critical thinking about media, are relevant for research examining media literacy in the context of eating disorder risk factors. *Media processing* has been outlined in the message interpretation process model [8] and adapted for application to eating disorder prevention research [6, 9]. The model specifies processes by which individuals interpret and internalise media messages and images, and ultimately, whether media messages and portrayals are accepted or rejected. These processes involve assessments of the *realism*, *similarity*, and *desirability* of media messages. Lower levels of scepticism in relation to media being interpreted as realistic, similar to one’s own and other’s experiences, and highly desirable, are proposed to lead to greater acceptance of media messages [8].

Consideration of these processes, particularly the realism of media images, is highly relevant for media literacy approaches to prevent body dissatisfaction and disordered eating. Indeed, evaluations of approaches that have highlighted the artificial and unrealistic nature of appearance ideals portrayed in media have demonstrated positive effects in preventing media induced body dissatisfaction [10, 11]. Thus, realism is an especially important construct to examine in relation to eating disorder risk factor research.

*Critical thinking*, a key element of media literacy, is thought to inform interpretation of media and facilitate independent and informed judgements about media [12]. According to the media literacy framework proposed by Primack and colleagues [4], critical thinking skills reflect an understanding of the purpose of media, including that values and points of view are portrayed in media messages, that media can impact attitudes and behaviours, that various techniques are used to produce media, and that media may, or may not, be a true representation of reality.

Critical thinking skills in relation to media messages that encourage acceptance and pursuit of appearance ideals have been identified as a fundamental component of media literacy-based interventions for eating disorder risk. For example, Wilksch and Wade’s Media Smart program [13, 14] promotes critical thinking to identify sources of media pressure, to recognise healthy and unhealthy messages from media, and to identify elements of media advertising that may be harmful to adolescents. In light of the crucial role that critical thinking plays in media literacy-based eating disorder prevention, establishing the psychometric properties of critical thinking measures is needed in order to effectively examine the role of critical thinking in change in eating disorder risk factors. Although the role of critical thinking has been examined in relation to other health risk behaviours [15–17] no published data exist that establish a relationship between critical thinking and eating disorder risk, or that demonstrate the psychometric properties of such measures in eating disorders risk factor research.

The aim of the current study was to examine the psychometric properties of six measures of media processing and critical thinking about media in early adolescent females. This target group was selected because early adolescent females are at particular risk in relation to the effects of internalisation of the thin-ideal [18] and media influence [19], and early adolescence is a key transition time in developing disordered eating [20–22].

The measures examined in this paper were the *media processing measures*, the Realism, Similarity, and Desirability subscales of the Media Attitudes Questionnaire [MAQ; 6]; and the Fake subscale of the Critical Processing of Beauty Images Scale [23]; and the *critical thinking measures*, the Critical Thinking about Media Messages scale (CTMM; [15]), and the Critical Thinking about Media Messages - Appearance Focus scale (CTMM-AF), the latter of which was adapted from the CTMM [24]. Measures were selected because of their applicability to constructs addressed in media literacy interventions and consequently, their potential ability to detect change in media processing and critical thinking skills that may result from media literacy interventions. In addition, the MAQ and Fake subscale had previously been used in body image research with adolescent and college age females [3, 6, 23, 25], and were brief, making them user friendly. Further, the CTMM has also been used previously with female adolescent samples [15], is brief, and is a general critical thinking measure without specific appearance-related content. In contrast, the CTMM-AF was designed to capture critical thinking, but with a focus on appearance-related media.

The current study aimed to examine the factor structure, internal consistency, test-retest reliability, and construct, convergent, and discriminant validity of scores on the six media processing and critical thinking measures, as well as to determine their suitability for use in eating disorders risk factor research. In regard to construct validity, it was expected that the media literacy measures that assess similar constructs would have stronger relationships among them, than with those that assess related but distinct constructs, e.g., media processing measures would be more strongly correlated with one another than media processing measures would be with critical thinking measures. To further demonstrate construct validity, consistent with the sociocultural model of body dissatisfaction and disordered eating, higher levels of media scepticism in relation to media processing and higher levels of critical thinking about media were expected to be related to lower levels of eating disorder risk factors. In addition, media literacy variables were expected to have stronger relationships with variables in the sociocultural model theorised to be proximally related, such as internalisation of the thin-ideal and appearance comparison, and weaker relationships with variables theorised to be distally related, such as body dissatisfaction and disordered eating.

## Method

### Participants

Secondary data from two separate studies were utilised in the current study [18, 24]. Two separate samples were included, each sample completing a different combination of media literacy questionnaires. Both samples comprised female grade 7 students (7^th^ year of schooling, similar to grade 7 in the US) recruited from secondary (i.e., high) schools in Melbourne, Australia to participate in separate studies of body image. Sample A [24], (*n* = 216, *M*_age_ = 12.99 years, *SD* = 0.40), was recruited from three single-sex independent schools. Sample B [18], (*n* = 208, *M*_age_ = 12.78 years, *SD* = 0.46) was recruited from four government schools (three co-educational and one single-sex). Most participants (84.3 and 82.2 % for samples A and B respectively) were born in Australia, with others born in a diverse range of countries.

### Measures

Participants completed self-report questionnaires. These were in online format for sample A and pen-and-paper format for sample B. Participants provided information on their age and country of birth.

#### Media processing and critical thinking variables

##### Media attitudes questionnaire

The MAQ was designed to assess media scepticism in relation to media images [6, 25]. Items were derived from the message interpretation process model, and thus, the MAQ is a media processing measure. Ten items, focused on the appearance of models in media, from the MAQ, a 22 item measure, were included. Four had been described in two studies [6, 25] as indicating desirability and six items had been described as indicating realism in one study of adolescents [6], although in a study of college age women those items were divided into realism (3 items) and similarity (3 items) subscales [25].

The 10 included items that corresponded to MAQ Realism, Similarity, and Desirability subscales [25] were used in the current study as these subscales reflected core aspects of the relevant media processing theory and they have been shown to significantly differ between control and intervention groups following participation in media literacy-based body image interventions [6, 25]. Three items were from the Realism subscale (for example “Normally women (in real life) are as thin as the models in ads”) which assesses the extent to which media images are perceived as realistic portrayals of social reality; three items were from the Similarity subscale (for example “I could be as thin as the models in ads”), which assesses the extent to which media images are perceived to be similar to oneself and others) and four items were from the Desirability subscale (for example “Models in ads have lots of fun”), which assesses the extent to which models shown in media are perceived to be desirable or attractive. Participants responded to items on a 5-point scale from 1 (*completely disagree*) to 5 (*completely agree*). Items were reverse scored prior to forming subscale scores so that higher scores reflected higher scepticism about media (subscales were thus relabelled Realism Scepticism, Similarity Scepticism, and Desirability Scepticism). Subscale scores were calculated from the mean of the responses to items from each subscale (range 1 to 5). Other than internal consistency, which has been shown to be in the low to adequate range (Cronbach’s alpha range: .56 to .92 [6, 25]), psychometric data for scores on this measure are not available. The MAQ was completed by participants from both samples.

##### Fake subscale

This media processing measure from the Critical Processing of Beauty Images scale [23], with items such as “That kind of perfection isn’t real” was designed to assess frequency of thoughts that media images depicting female models are artificially created and unrealistically perfect. Participants responded to five items on a 5-point scale from 1 (*never*) to 5 (*always*). A total score (range 5 to 25) was calculated from the sum of items. Higher scores reflect greater frequency of critical processing of images. Scores on the Fake subscale in college age females have been shown to have high internal consistency (α = .92) and adequate test-retest reliability over intervals of one, two, three, and four weeks (range: *r* = .67 to *r* = .86) [23]. Construct validity was demonstrated with significant positive associations between scores on the Fake subscale and the number of critical thoughts generated following viewing of thin-ideal appearance advertising images. However, the Fake subscale was not correlated with measures of body image, internalisation of the thin or athletic ideal, or perceived pressure from media [23]. This measure was completed by participants from sample B.

##### Critical thinking about media messages

The six items of the CTMM scale [15] assess the frequency with which participants think critically about media messages. Participants respond to items such as “I think about what the people who made the media message want me to believe” on a 6-point scale from 1 (*never*) to 6 (*always*). A total score (range 6 to 36) was calculated from the sum of item responses with higher scores reflecting greater frequency of critical thinking about media messages. Items from this measure reflect critical thinking in relation to each of the domains of the media literacy framework [4]. Scores for this measure have demonstrated high internal consistency in pre- to mid-adolescent samples ranging in age from 10 to 15 years (α = .92) [15]. The CTMM was completed by participants in sample A.

##### Critical thinking about media messages – appearance focus

The six items of the CTMM-AF scale assess the frequency with which participants think critically about appearance-related media messages. The items for this scale were modified from the CTMM scale [15, 24]. Consistent with the original scale, this adapted measure contains items that represent critical thinking in relation to each of the media literacy domains [4]. Responses to items such as “When I look at ads with thin female models I think about what the people who made the media message want me to believe” were indicated on a 6-point scale from 1 (*never*) to 6 (*always*). A total score (range 6 to 36) was calculated from the sum of item responses with higher scores reflecting greater frequency of critical thinking about appearance focused media. Psychometric data for this measure are not available. The CTMM-AF was completed by participants from sample A.

##### Measures for convergent validity

Measures of media use and eating disorder risk factor variables were completed by participants in both samples.

##### Media exposure

The average number of hours per day exposed to screen-based media for non-school or homework related activities was assessed with the Screen Habits scale [26, 27]. Participants from sample B responded to two items to indicate how many hours on weekdays and weekend days they spend using computers and watching television and DVDs. Participants from sample A responded to these two items and an additional item to indicate how many hours they spend using smart phones and tablets. A weighted score to account for weekday and weekend exposure was calculated from the average daily media use. Scores ranged from zero to 10 h for sample B and zero to 15 h for sample A, with higher scores reflecting higher levels of media exposure. In adolescent samples, scores on this measure have demonstrated adequate test-retest reliability and construct validity [26, 27].

##### Body dissatisfaction

The nine items of the Body Dissatisfaction subscale of the Eating Disorders Inventory (EDI-2; [28]) were completed by participants from both samples. Participants responded to items such as “I think that my thighs are too large” on a 6-point scale from 0 (*never*) to 5 (*always*). Item responses were summed to form a total score (range 0 to 45) with higher scores reflecting higher body dissatisfaction. Scores on this measure have demonstrated high internal consistency [29] and validity in adolescent samples [28]. Internal consistency for the current study was α = .91 and α = .91 for samples A and B respectively.

##### Internalisation of the thin-ideal

The internalisation-general subscale of the Sociocultural Attitudes Towards Appearance Questionnaire -3 [30] assessed the extent to which participants internalised sociocultural standards for appearance as personal standards. All nine items were used in sample B. Three items were removed for sample A to reduce participant burden. Responses to items such as “I would like my body to look like the people who are on TV” were indicated on a 5-point scale from 1 (*strongly disagree*) to 5 (*strongly agree*). The score range was 5 to 30 for sample A and 5 to 45 for sample B. Higher scores reflect higher internalisation of the thin-ideal. Scores have shown high internal consistency in young adolescent girls [31] and college age females, and convergent validity in college age samples [30]. Internal consistency for the current study was α = .92 and α = .95 for samples A and B, respectively.

##### Appearance comparison

The five item Physical Appearance Comparison Scale [32] assessed frequency of appearance comparison. Participants responded to items such as “I compare my looks to the looks of others to determine if I am attractive or unattractive” on a 5-point scale from 1 (*never*) to 5 (*always*). A total score (range 5 to 25) was calculated from the sum of item responses. Higher scores reflect higher frequency of appearance comparison. In early adolescent female samples, scores for this scale have previously shown high internal consistency [29]. In older adolescent female samples (grade 10), test-retest reliability has been shown to be adequate [33]. In college age women, scores on the Physical Appearance Comparison Scale have correlated highly with other appearance comparison measures [34], supporting construct validity. Internal consistency for the current study was α = .92 and α = .88 for samples A and B, respectively.

##### Dietary restraint

The 10-item Restrained Eating subscale of the Dutch Eating Behavior Questionnaire [35] assessed dietary restraint. Participants responded to items such as “Do you deliberately eat less in order not to become heavier?” on a 5-point scale from 1 (*never*) to 5 (*very often*). A total score (range 1 to 5) was calculated from the mean of item responses with higher scores reflecting higher dietary restraint. Internal consistency and test-retest reliability of scores on this measure have been shown to be high in female adolescent samples with mean age 14.5 years (*SD* = 0.53) [36]. Internal consistency for the current study was α = .94 and α = .95 for samples A and B, respectively.

### Procedure

Approvals were received from the La Trobe University Human Ethics Committee and state Education Department. Written parental informed consent and participant assent was provided by all participants. No student participants for whom parental consent was provided declined to take part. Participants from sample A completed the measures on one occasion (Time 1) and participants from sample B completed the measures on two occasions (time 1 and time 2) separated by seven weeks. Participants from both samples completed all of the measures for convergent validity: media exposure, thin-ideal internalisation, appearance comparison, body dissatisfaction, and dietary restraint. Both samples were also asked to complete the MAQ. Participants from sample A also completed the CTMM and CTMM-AF and participants from sample B completed the Fake scale. An additional separate test-retest sample of participants (*n* = 30; *M*_*age*_ = 13.10, *SD* = .04) completed only the CTMM and CTMM-AF on two occasions, separated by four weeks. Questionnaire completion took place in school classrooms, supervised by a researcher and a class teacher.

### Data analysis

To verify the factor structure of the Fake, CTMM and CTMM-AF, items for each scale were subject to Confirmatory Factor Analyses (CFA) using SPSS AMOS (version 22) conducted separately within sample A (CTMM and CTMM-AF) and sample B (Fake; time 1 assessment). The models were specified on the basis of the factor structure of the published scales. Model fit was assessed with chi-square, for which small, non-significant values indicate good fit, Confirmatory Fit Index, Tucker-Lewis Index (CFI, TLI; ≥.95 indicates good fit), and Root Mean Square Error of Approximation (RMSEA; ≤ .05 indicates good fit, ≤.08 indicates adequate fit) [37, 38].

Previous use of the MAQ was inconsistent in relation to specification of subscales and the factor structure had not previously been examined with exploratory or confirmatory factor analyses. Thus, in sample A, the MAQ was initially subject to exploratory Principal Components Analysis (PCA), using oblique (Direct Oblimin) rotation to account for potentially correlated components, to identify the factor structure. The best fitting structure was then subject to confirmation in sample B with CFA. Measurement invariance of the factor structure across samples A and B was conducted with multi-group analysis in AMOS and chi-square differences were examined with a statistical calculator [39].

Internal consistency was calculated using Cronbach’s alpha or, for two-item scales, the Spearman-Brown coefficient [40]. Construct reliability (CR) values were also obtained from CFA output to provide further indication of internal consistency. Values of ≥.70 indicate acceptable internal consistency [41, 42]. Test-retest reliability was assessed with intra-class correlation coefficients (ICC) for agreement (two-way random effects model). The criterion for adequate test-retest reliability was a coefficient of ≥.70 [43].

Construct validity was assessed by examining zero-order correlations between the six measures of media processing and critical thinking as well as between the media literacy measures and eating disorder risk factor variables. Steiger’s tests [44] to compare the strength of dependent correlations were conducted using an online calculator [45]. The average variance extracted (AVE) values calculated from CFA output were also examined, with values >.50 indicating adequate item convergent validity [42]. Discriminant validity between constructs was assessed by simultaneously modelling with CFA scales for which data was available in each sample. Discriminant validity is indicated where the AVE value for each construct is greater than the square of the correlations between constructs [42].

Sample size differed slightly for sample B (range *n* = 201 to 208) due to sporadic missing data for a few individual questions. Due to the mostly cross-sectional nature of the analyses, data were not imputed to account for missing data.

## Results

### Descriptive statistics

Means, standard deviations, and scale ranges for each media processing and critical thinking measure in samples A and B are shown in Table 1. The means for the measures were relatively consistent across samples.

**Table 1:** Descriptive Statistics, Internal Consistency Reliability and Test-retest Reliability for Media Processing and Critical Thinking Variables

	Scale range	Sample A (*N* = 216)			Sample B (*N* = 208)				
		Time 1			Time 1			Time 2	
		Mean (SD)	α	CR	Mean (SD)	α	CR	Mean (SD)	ICC
Media attitudes questionnaire (MAQ)									
Realism scepticism subscale	1 to 5	4.13 (0.94)	.80 ^a^	.80	3.80 (1.06)	.87	.86	4.10 (0.99)	.64***
Similarity scepticism subscale	1 to 5	3.49 (1.12)	.83	.85	3.37 (1.00)	.76	.79	3.49 (1.04)	.81***
Desirability scepticism subscale	1 to 5	2.90 (1.03)	.82	.84	2.94 (0.98)	.79	.80	3.16 (1.04)	.70***
Critical thinking about media messages	6 to 36	17.56 (6.91)	.90	.90	NA	NA	NA	NA	NA
Critical thinking about media messages – Appearance focus	6 to 36	15.78 (7.94)	.95	.95	NA	NA	NA	NA	NA
Fake subscale	1 to 5	NA	NA	NA	3.04 (0.99)	.92	.93	3.06 (1.07)	.80***

*Note. α* Cronbach’s alpha, *CR* construct reliability, *ICC* intraclass correlation coefficient, *NA* data not available

*p < .05; **p < .01; ***p < .001

^a^Spearman-Brown coefficient calculated for internal consistency of a two-item scale

### Factor structure

#### MAQ

The PCA of the ten items from the MAQ in sample A revealed three factors with eigenvalues greater than one. Inspection of the scree plot revealed a clear break after the first component, and a weak break after the third component. Parallel analysis supported retention of three factors. Following oblique rotation, the majority of the 10 items loaded strongly and separately on the three components (see Table 2), with the exception of items 3 (the models in advertisements are real people) and 7 (models in ads are intelligent) which had low item loadings, and also loaded across each of the three components with none of the loadings >.40. In addition, the communality values for both item 3 and 7 were relatively low (.42 and .38, respectively). Items 3 and 7 were omitted.

**Table 2:** Items and Factor Loadings for the Media Attitudes Questionnaire

	Component loading from PCA^a^			Factor loading from CFA^b^		
Item (original subscale designation [6, 25])	1. Similarity scepticism	2. Realism scepticism	3. Desirability scepticism	1. Similarity scepticism	2. Realism scepticism	3. Desirability scepticism
1. Normally women (in real life) look like models in ads (Ad, Realism; C, Realism)	-.02	**.92**	.05		.89	
2. Normally women (in real life) are as thin as the models in ads (Ad, Realism; C, Realism)	-.06	**.90**	.00		.86	
3. The models in advertisements are real people (Ad, Realism; C, Realism)	.40	.33	-.07	-	-	-
4. I could look like the models in ads (Ad, Realism; C, Similarity)	**.94**	-.05	.06	.84		
5. I could be as thin as the models in ads (Ad, Realism; C, Similarity)	**.97**	-.10	.03	.90		
6. Most women could be as thin as the models in ads by exercising and/or dieting (Ad, Realism; C, Similarity)	**.57**	.16	-.22	.47		
7. Models in ads are intelligent (Ad, Desirability; C, Desirability)	.23	.33	-.25	-	-	-
8. Models in ads are beautiful (Ad, Desirability; C, Desirability)	.01	-.06	**-.88**			.73
9. Models in ads have perfect bodies (Ad, Desirability; C, Desirability)	-.01	-.06	**-.92**			.94
10. Models in ads have lots of fun (Ad, Desirability; C, Desirability)	-.04	.07	**-.80**			.53
Average variance extracted				.57	.76	.59

Major loadings on components are in bold

*Note. PCA* principal components analysis, *CFA* confirmatory factor analysis, *Ad* adolescent sample [6], *C* college sample [25]

^a^Sample A only (*N* = 216); ^b^Sample B only (*N* = 201)

Data from sample B were subjected to CFA using the PCA in sample A to identify the model to be tested. Three items (4, 5, and 6) were specified for factor 1 (Similarity Scepticism), two items (1 and 2) for factor 2 (Realism Scepticism), and three items (8, 9, 10) for factor 3 (Desirability Scepticism). The CFA analysis revealed adequate fit to the data, *χ* ^2^(13) = 27.77, *p* = .010, CFI = .98,TLI = .95, RMSEA = .08. Three items were retained for the Similarity Scepticism subscale, two were retained for the Realism Scepticism subscale, and three were retained for the Desirability Scepticism subscale as per the component structure identified from the PCA in sample A. Factor loadings are shown in Table 2.

##### Invariance testing

Multi-group analysis of the items of the MAQ was conducted with sample A and sample B to test for invariance of the identified factor structure between samples. The unconstrained *χ* ^2^(13) = 50.76, *p* < .001, and constrained *χ* ^2^(34) =76.30, *p* < .001 models were not significantly different *χ* ^2^(21) = 25.4, *p* = .230, indicating that the identified factor structure was invariant across sample A and sample B.

#### CTMM

The CFA of the 6 items from the CTMM revealed good fit to the data, *χ* ^2^(7) = 9.06, *p* = .327, CFI = 1.00, TLI = 1.00, RMSEA = .03. The model fit in this sample supported the one factor structure of the original scale.

#### CTMM-AF

The CFA of the 6 items from the CTMM-AF also revealed good fit to the data, *χ* ^2^(7) = 11.24, *p* = .129, CFI = 1.0, TLI = .99, RMSEA = .05. The one factor structure for the CTMM-AF was also supported in this sample.

#### Fake

The CFA of the 5 items of the Fake subscale showed good fit to the data, *χ* ^2^(2) = 1.08, *p* = .406, CFI = 1.00, TLI = 1.00, RMSEA = .00. The one factor structure of the Fake subscale was supported in this sample. Items and factor loadings from CFA outcomes for the CTMM, CTMM-AF, and Fake subscale are shown in Table 3.

**Table 3:** Items and Factor Loadings for the CTMM, CTMM-AF, and Fake Scales

Measure and item	Factor loading
Critical Thinking about Media Messages^a^	
3. I think about what the people who made the media message want me to believe	.90
4. I think about the things the advertisers do to get my attention	.82
2. I think about who created the message I see on the ad	.73
1. I think about the purpose behind a message I see on television	.71
5. I think about whether the things that advertisers want me to do are good for me	.71
6. I try and think about how true or false an advertisement is	.68
Average variance extracted	.60
Critical Thinking about Media Messages – Appearance Focus^a^	
4. When I see ads with very attractive female models I think about the things the advertisers do to get my attention	.92
3. When I look at ads with thin female models I think about what the people who made the media message want me to believe	.91
2. When I see very attractive female models used in ads I think about who created the message I see on the ad	.90
1. When looking at thin female models on television I think about the purpose behind the message I see	.87
5. When looking at ads with thin female models I think about whether the things that advertisers want me to do are good for me	.83
6. When I see ads about ways to be more attractive I try and think about how true or false an advertisement is	.75
Average variance extracted	.76
Fake^b^	
3. They probably used computer re-touching to make her look like that	.92
2. Nobody looks like that without computer tricks	.90
5. It takes a lot of camera tricks to make someone look that good	.84
4. That kind of perfection isn’t real	.81
1. She’s airbrushed	.68
Average variance extracted	.71

^a^Sample A only (*N* = 216); ^b^Sample B only (*N* = 204)

### Internal consistency reliability

Cronbach’s alpha and Spearman-Brown coefficient values, as well as construct reliability values for internal consistency of measures are shown in Table 1. All measures, in both samples, showed adequate to excellent internal consistency of items.

### Test-retest reliability

Test-retest reliabilities of the MAQ and Fake subscales were examined in sample B and for the CTMM and CTMM-AF in the test-retest only sample. Intra-class correlation coefficients, shown in Table 1, indicated adequate agreement (≥.70) between Time 1 and Time 2 scores for the MAQ and Fake subscales, with the exception of the Realism Scepticism subscale where agreement was slightly below the adequate threshold. In the test-retest only sample, scores on the CTMM (*ICC* = .78, *p* < .001, *M*_*T1*_ = 14.50, *SD*_*T1*_ = 5.73, *M*_*T2*_ = 15.41, *SD*_*T2*_ = 7.15) and CTMM-AF (*ICC* = .70, *p* = .001, *M*_*T1*_ = 13.77, *SD*_*T1*_ = 5.98, *M*_*T2*_ = 13.47, *SD*_*T2*_ = 5.97) demonstrated adequate test-retest reliability.

### Construct validity

As shown in Table 4, moderate positive correlations between the media processing variables Realism Scepticism, Similarity Scepticism, and Desirability Scepticism were revealed in both samples, indicating convergent validity. The Fake subscale, also considered to be a media processing variable was only correlated with Realism Scepticism and the correlation was of small magnitude. The two critical thinking variables, CTMM and CTMM-AF were very strongly correlated with one another in sample A, demonstrating convergent validity. The related but different types of media literacy variables, media processing and critical thinking, did not correlate strongly, the highest correlation being *r* = .14 between CTMM and both Realism Scepticism and Similarity Scepticism. The difference between the magnitude of the correlations was confirmed with Steiger’s tests [44] using Lee and Preacher’s software [45]. Correlations between media processing variables, Realism Scepticism, Similarity Scepticism, and Desirability Scepticism, and correlations between critical thinking variables, CTMM and CTMM-AF, were significantly stronger than correlations between any combination of media processing and critical thinking variable (all *z*s >2.2, all *p*s <.02).

**Table 4:** Bivariate Correlations between Media Processing and Critical Thinking Variables

	1	2	3	4	5	6
*Sample A*						
1. Realism scepticism (MAQ)	-	.36***	.34***	.14*	.10	NA
2. Similarity scepticism (MAQ)		-	.47***	.14*	.06	NA
3. Desirability scepticism (MAQ)			-	.07	.04	NA
4. Critical thinking about media messages				-	.80***	NA
5. Critical thinking about media messages - Appearance focus					-	NA
*Sample B*						
1. Realism scepticism (MAQ)	-	.23**	.38***	NA	NA	.15*
2. Similarity scepticism (MAQ)		-	.28***	NA	NA	-.01
3. Desirability scepticism (MAQ)			-	NA	NA	.05
6. Fake				NA	NA	-

*Note. MAQ* media attitudes questionnaire, *NA* data not available

*p < .05; **p < .01; ***p < .001

Item-level convergent validity was further supported by AVE values (see Tables 2 and 3) which for all measures were above the threshold of .5 for adequate convergence [42]. Discriminant validity between media processing and critical thinking variables was examined by comparing the AVE values for each construct with the square of the correlations between constructs. For all pairs of constructs, with the exception of the CTMM compared with CTMM-AF, the AVE value was greater than the squared correlation, supporting discriminant validity. The AVE value for CTMM was smaller than the squared correlation between CTMM and CTMM-AF, indicating that the CTMM did not have discriminant validity from the CTMM-AF. Discriminant validity for measures in sample B was also examined by comparing AVE values with the square of the correlations. For all pairs of constructs, the AVE value was greater than the squared correlation, supporting discriminant validity between MAQ subscales and between the MAQ subscales and the Fake subscale.

Correlations were calculated between the media processing and critical thinking variables and the eating disorder risk factor variables to examine further evidence for construct validity (see Table 5). A number of significant associations were revealed. The majority of observed relationships were in the expected direction such that higher levels of media scepticism for MAQ subscales, and higher levels of critical thinking for CTMM and CTMM-AF, were associated with lower levels of eating disorder risk factor variables. The Fake subscale was not significantly correlated with any of the eating disorder risk factor variables. Unexpectedly, in sample B, a positive correlation was revealed between Similarity Scepticism and body dissatisfaction, indicating that high similarity scepticism scores were related to high body dissatisfaction. The direction of this relationship was inconsistent with other observed relationships.

**Table 5:** Bivariate Correlations between Media Literacy Variables and Eating Disorder Risk Factor Variables

	Age	Media exposure	Internalisation of the thin-ideal	Appearance comparison	Body dissatisfaction	Dietary Restraint
*Sample A*						
Realism scepticism (MAQ)	-.01	-.06	-.27***	-.13*	-.14*	-.28***
Similarity scepticism (MAQ)	-.03	-.09	-.29***	-.14*	-.04	-.36***
Desirability scepticism (MAQ)	-.05	-.13****	-.53***	-.32***	-.27***	-.37***
Critical thinking about media messages	-.07	-.21**	-.13*	.00	-.13****	-.02
Critical thinking about media messages - Appearance focus	-.04	-.18**	-.09	.03	-.12	-.01
*Sample B*						
Realism scepticism (MAQ)	.23**	.08	-.40***	-.15*	-.07	-.13****
Similarity scepticism (MAQ)	.04	-.02	-.19**	-.09	.25***	.09
Desirability scepticism (MAQ)	.00	-.10	-.45***	-.38***	-.18*	-.19**
Fake	-.01	-.13****	-.01	.10	-.02	.07

*Note. MAQ* media attitudes questionnaire, *NA* data not available

*p < .05; **p < .01; ***p < .001; ****p < .10

Results from Steiger’s tests comparing the magnitude of relationships between media literacy variables and eating disorder risk factor variables that were expected to be proximally related (internalisation of the thin-ideal and appearance comparison) with the magnitude of relationships between media literacy variables and eating disorder risk factor variables that were expected to be distally related (body dissatisfaction and dietary restraint) were mixed. For example, in Sample A, Realism Scepticism had a stronger relationship with internalisation of the thin-ideal than with body dissatisfaction (*z* = −2.20, *p* = .014) but the former relationship was not stronger than the relationship between Realism Scepticism and dietary restraint (*z* = 0.16, *p* = .438). Also in Sample A, the relationship between CTMM and internalisation of the thin-ideal was stronger than the relationship between CTMM and dietary restraint (*z* = −1.65, *p* = .049), whereas the relationship between CTMM and appearance comparison was not stronger than the relationship between CTMM and dietary restraint (*z* = 0.29, *p* = .385). Results from all comparisons are available from the authors upon request.

Age was not related to the media literacy variables with the exception of a positive relationship with Realism Scepticism in sample B showing that older age was associated with higher scepticism. Variables assessing the media processing constructs were not significantly related to media exposure. The critical thinking variables both had small inverse correlations with media exposure such that lower levels of exposure to screen-based media were associated with higher levels of critical thinking.

## Discussion

The aim of this study was to examine the psychometric properties of six measures of media processing and critical thinking about media. The findings provided support for the factor structure, internal consistency, test-retest reliability, and construct validity of Realism Scepticism, Similarity Scepticism, Desirability Scepticism, and Fake subscales, and the CTMM and CTMM-AF in early adolescent female samples in the context of eating disorder risk factor research.

### Factor structures

In line with the original scale development, CFA confirmed each of the one-factor structures of the Fake [23], CTMM, and CTMM-AF scales [15]. These brief measures clearly each assess one construct, with each item from the scales loading highly on their respective measures. The best fitting model for the MAQ, which was invariant across both samples, deviated slightly from the original measure [6, 25]. Although the general factor structure was supported, two items (one from Realism Scepticism and one from Desirability Scepticism) were removed to improve model fit.

The omission of these items raises questions about what the excluded items assess. The omission of “The models in advertisements are real people” from the Realism Scepticism subscale perhaps resulted from lack of clarity in the wording of the item. Anecdotal feedback from participants during data collection indicated that this item caused confusion for some participants in relation to what the term “real” referred. This confusion may have led to inconsistent responding and contributed to poor model fit. Simplifying the wording of the item, for example “The models in advertisements look unrealistic”, would better ensure that participants understood that the term *real*, or *unrealistic* in the reworded example, refers to the appearance of models in media.

It seems clear from the Desirability Scepticism subscale that the omitted item (Models in ads are intelligent) differs from the remaining items, which focus on perceptions of models as being beautiful, having perfect bodies, and having fun. These latter attributes are much simpler to infer from visual images than a judgement about intelligence, and are also features that are much more likely to be portrayed in images containing models whose appearance is consistent with the thin-ideal, which are prominent in contemporary media [46]. Thus, the perceptions of intelligence item may be less relevant for assessing desirability of media in relation to appearance and eating content.

### Internal consistency and test-retest reliability

The media processing and critical thinking measures demonstrated adequate to excellent internal consistency and test-retest reliability. Levels of internal consistency in the current sample were mostly consistent with previous research, particularly for the Fake subscale [23] and the CTMM [15]. Values for Realism Scepticism, Similarity Scepticism, and Desirability Scepticism scales were higher in the current study than previously published [6, 25].

Published test-retest reliability information was available only for the Fake subscale [23] and was comparable to the high level of consistency found in the current study. The findings of this study have established support for the reproducibility of scores of media processing and critical thinking about media measures in early adolescent girls, which was not previously available.

### Construct validity

The relationships between the measures of media literacy-related constructs provided support for the construct validity of scores on these measures. The media processing variables, Realism Scepticism, Similarity Scepticism, and Desirability Scepticism had moderate correlations with one another, although the Fake subscale was correlated only with Realism Scepticism. The measures of critical thinking were highly correlated with one another. As the CTMM-AF was derived from the CTMM and contains items with very similar wording, the lack of discriminant validity between the two measures was not surprising. Further examination of the construct validity of these measures in relation to other types of critical thinking measures is needed.

The two types of measures, media processing and critical thinking, were shown to be somewhat related to one another, with positive correlations between both Realism Scepticism and Similarity Scepticism, and the CTMM. The smaller magnitude of these correlations, in comparison to the correlations between measures of the same constructs, as well as the results from the discriminant validity analyses, supports the contention that media processing and critical thinking are somewhat related, but distinct constructs. Interestingly, the CTMM-AF, whose items had appearance-related content, did not have stronger relationships than the non-appearance focused CTMM with the MAQ subscales which also have appearance-related content. This suggests that it is the processing and critical thinking components that are related, rather than the focus on appearance that contributes to the shared variance. Further research to establish the common and distinct feature of these types of measures will enhance our understanding of the effects of media literacy on eating disorder risk factors.

The pattern of relationships between media literacy related variables and eating disorder risk factors partially supports the theoretical framework of the role of media literacy in eating disorder risk as outlined above and hence contributed further to evidence for construct validity for these measures. As predicted, higher levels of media scepticism and critical thinking about media were associated with lower levels of eating disorder risk in relation to internalisation of the thin-ideal, appearance comparison, body dissatisfaction, and dietary restraint, although relationships were of larger magnitude for subscales of the MAQ than for the Fake subscale and both of the critical thinking variables, CTMM and CTMM-AF. Further, the magnitude of the correlations between media processing variables and internalisation of the thin-ideal and appearance comparison, which based on the sociocultural model were expected to be proximally related to media literacy, were stronger than the correlations with body dissatisfaction, predicted to be distally related to media literacy. In sample A, the strong association between media processing variables and dietary restraint, a variable predicted to be distally related to media processing, did not, however, fit this expected pattern. Further examination will clarify if a direct relationship to body dissatisfaction and dietary restraint exists, or if the relationship is mediated by internalisation of the thin-ideal and appearance comparison as proposed in the theoretical framework above.

The critical thinking measures were inversely associated with media exposure. Although the effect was small, it suggests that the more one thinks critically of media, the less one engages with the medium. The temporal sequence of this relationship, is however, unknown.

The relationships described are consistent with the contention that media literacy reduces the persuasive impact of media, thereby diminishing the levels of relevant sociocultural eating disorder risk factors [3, 9]. Additional evidence from prospective and experimental studies is required to examine and confirm the temporal sequences of these relationships, i.e., to confirm that increases in media literacy skills precede reductions in appearance comparison and internalisation of appearance ideals. Such evidence will contribute to support for the predictive validity of media literacy concepts in predicting change over time in variables theorised to be proximally related to media literacy.

Somewhat surprisingly, the relationships between the Similarity Scepticism scale and body dissatisfaction was negligible in sample A and positive in sample B. It is difficult to reconcile these findings but one may speculate that participants interpreted items differently, affecting observed relationships. It is possible that some participants interpret low similarity of self to media (high Similarity Scepticism scores) as the self not meeting media standards for appearance, whereas for others, low similarity to media reflects high scepticism of the likelihood of self (and others) meeting the unrealistic appearance standards of media. In this manner, the former interpretation would be associated with high body dissatisfaction, whereas the latter would not. It is unclear why one group of participants would interpret these items in one way and another in a different manner, although it seems unlikely that the differences in school types, co-educational versus single-sex, or in data collection procedure, online versus paper, would have affected the interpretation of items. It may also be the case that additional influences on body dissatisfaction that were not assessed in this study, such as perfectionism, moderate the relationship between similarity scepticism and body dissatisfaction. In light of these findings, caution should be used with this scale and resolution of the contrasting relationships through replication and examination of moderating variables is warranted.

The Desirability Scepticism scale was found to correlate strongly with internalisation of the thin-ideal but lacked shared variance with measures of critical thinking. These findings, considered alongside an examination of the content of items of the Desirability Scepticism scale, for example “Models in ads have perfect bodies”, suggest that this scale is less about scepticism of media, and more about endorsement of models in media. Such a conceptualisation would explain the high correlation with internalisation of the thin-ideal, as previous research has shown that women who perceive positive rewards from media appearance ideals had higher levels of internalisation of the thin-ideal [47]. While assessing endorsement of media portrayals is a useful concept for eating disorder risk factor research, particularly in regard to research examining media influences on body image, further work is required to clarify if this concept is consistent with a media literacy framework in relation to scepticism or critical thinking about media.

In regard to the Fake subscale, significant correlations were revealed only with Realism Scepticism and the correlation was small. No relationships were observed between the Fake subscale and any of the eating disorder risk factor variables, consistent with previous research [23]. Notwithstanding the high internal consistency and test-retest reliability for scores on this measure in both the current study and in previous research [23], the lack of support for construct validity for the Fake subscale calls into question its utility in assessing media literacy processing in eating disorder risk factor research.

### Limitations

This study had a number of limitations. Outcomes of the examination of the factor structure of the subscales of the MAQ resulted in a two-item scale for realism scepticism. Although two-item scales are parsimonious, and were clearly indicated as the best fit to the data from the current analyses, further expansion of this measure could be useful. In relation to the CTMM and CTMM-AF, examination of construct validity against independent measures of critical thinking was not conducted in the current study. In addition, the psychometric properties were tested in early adolescent female samples and replication in diverse samples is required. The strengths of this study were the examination of multiple psychometric properties of six different measures of media processing and critical thinking about media literacy. The use of two separate samples strengthened the findings, although it would have been advantageous to have assessed all measures in both samples which would have allowed all scales to have been tested in one model.

## Conclusions

The results of this study provide the first evidence in female adolescent samples for the factor structure, internal consistency and test-retest reliability, and construct validity of scores from media processing and critical thinking media literacy measures: Realism Scepticism, Similarity Scepticism, Desirability Scepticism, CTMM, and CTMM-AF. Evidence for the construct validity of the Fake subscale was lacking. The other central aim of the study was to examine the suitability of measures of media literacy for use in eating disorders risk factor research. In light of the findings, the Realism Scepticism subscale and CTMM are recommended for use in future research. These two scales demonstrated adequate psychometric properties, had relationships with eating disorder risk factor variables that were consistent with predictions and supportive of media literacy theories, were related to but distinct from one another, and had face validity (e.g., compared with the Desirability Scepticism subscale as noted above). Thus, both scales would be suitable for research examining media literacy in relation to eating disorders risk factors and also for evaluation of media literacy-based prevention of body dissatisfaction and disordered eating. Future research may further improve the utility of the Realism Scepticism subscale by expanding the number of items that assess this construct. In light of the growing enthusiasm for implementation of media literacy-based interventions for preventing and reducing eating disorder risk, the study findings demonstrating preliminary psychometric properties of suitable media literacy measures will offer researchers suitable options for evaluating the effects of interventions on change in media literacy.
